# A colloidal gold immunochromatographic test strip based on McAbs anti-S protein to detect porcine epidemic diarrhea virus

**DOI:** 10.1128/spectrum.03387-25

**Published:** 2026-04-23

**Authors:** Chaofan Liu, Wei Wang, Feiyan Wang, Jialu Hou, Qingbo Shi, Qinye Song, Chen Yuan

**Affiliations:** 1 College of Veterinary Medicine, Hebei Agricultural University, Veterinary Biological Technology Innovation Center of Hebei Province 74562, Baoding, China; 2 Hanzhong Animal Disease Prevention and Control Center, Hanzhong, Shanxi, China; Children's National Hospital, George Washington University, Washington, DC, USA

**Keywords:** S1, monoclonal antibody, colloidal gold immunochromatographic test strip, PEDV

## Abstract

Porcine epidemic diarrhea (PED) is a highly contagious intestinal disease caused by the porcine epidemic diarrhea virus (PEDV), resulting in considerable economic losses for the global swine industry. Rapid diagnosis of PEDV is essential to develop effective prevention and control measures. The PEDV S1 protein serves as an efficient target for PEDV diagnosis. In this study, we successfully produced three monoclonal antibodies (McAbs) specific against the PEDV S1 protein, namely, H6, G3, and F2. ELISA showed that McAb F2 was IgG2a, McAbs H6 and G3 were IgG1, and the antigen site of McAb F2 was different from those of the other two antibodies. A colloidal gold immunochromatographic strip for PEDV detection was developed. McAb F2 conjugated with colloidal gold served as the detector antibody, McAb H6 was used as the capture antibody at the test line, and goat anti-mouse IgG was applied at the control line. The strip can specifically detect PEDV antigen without cross-reacting with TGEV, PCV2, PRV, PRRSV, or CSFV. It demonstrated high sensitivity with a detection limit of 1.2 TCID_50_/μL. Compared with reverse transcription RT-PCR, the coincidence rate of the two methods was as high as 96.1%. In summary, the colloidal gold immunochromatographic test strip based on PEDV S1 protein McAbs is a fast, sensitive, and accurate method for PEDV detection and epidemic monitoring.

**IMPORTANCE:**

Rapid diagnosis of PEDV is crucial to reduce economic losses in the swine industry. In this study, producing three monoclonal antibodies against the PEDV S1 protein enables the development of a colloidal gold immunochromatographic strip. This strip can specifically detect PEDV with high sensitivity and no cross-reaction with other viruses. Its high coincidence rate with reverse transcription RT-PCR proves its accuracy. The strip offers a fast, sensitive, and precise method for PEDV detection, which is crucial for timely prevention and control, thereby reducing economic losses in the global swine industry.

## INTRODUCTION

Porcine epidemic diarrhea virus (PEDV) is a member of the genus Alphacoronavirus within the coronavirus family and causes porcine epidemic diarrhea (PED). This condition is distinguished by the development of enteritis, the production of watery diarrhea, and the occurrence of vomiting, ultimately leading to severe dehydration and significant electrolyte imbalance (1). Since its initial identification in the United Kingdom in 1977, PEDV has spread extensively across the globe, exerting devastating impacts on the international swine industry, notably in regions such as North America and Asia (2, 3). The mortality rate of piglets infected with PEDV during their first week of life can be as high as 100% (4). Rapid and accurate PEDV diagnosis is essential for the effective design and implementation of prevention and control strategies (5, 6). Therefore, establishing a rapid and simple PEDV detection method is of great significance for the early control of PEDV outbreaks.

The genome of PEDV spans approximately 28 kilobases (kb) in length and encompasses seven open reading frames (ORFs), arranged from the 5′ to the 3′ end. These ORFs encode four structural proteins: spike (S), membrane (M), envelope (E), and nucleocapsid (N) proteins. In addition to these, ORF1a and ORF1b encode polyproteins that are processed into multiple nonstructural proteins essential for transcription and translation, while ORF3 encodes another nonstructural protein involved in these processes (7). Notably, the S protein plays a pivotal role in the virus’s entry into host cells. It assumes a rod-like, trimeric spike structure with a length of approximately 20 nm on the surface of viral particles (8). Based on the cleavage site of the furin protease and homology with other coronaviruses, the S protein can be subdivided into two domains, S1 (spanning amino acids 1–789) and S2 (amino acids 790–1,383) (9). Specifically, S1 primarily functions in receptor binding, thereby facilitating viral attachment, while S2 is responsible for promoting viral membrane fusion (10, 11). Numerous B-cell epitopes, capable of inducing neutralizing antibodies, have been identified within the S1 domain (12–15).

Vaccination represents an efficacious strategy for the prevention and control of PEDV; however, the immune response elicited by currently available inactivated and attenuated vaccines remains suboptimal (16–19). Rapid and accurate diagnosis of PEDV is a prerequisite for the design and implementation of prevention and control strategies. Recent research endeavors have delved into the development of an ELISA method aimed at detecting PEDV (16). This approach holds promise for assessing the vaccine’s effectiveness and the immune responsiveness of neonatal piglets against the virus. However, because ELISA requires skilled operation and specialized equipment, it may be impractical in some settings. In this context, colloidal gold immunochromatography emerges as a superior alternative for rapid screening and diagnosis of PEDV in pig farms or grassroots units. This technique boasts simplicity, speed, sensitivity, and efficiency in detecting the virus, thereby facilitating timely and accurate disease management (20).

To establish a diagnostic tool capable of rapid and precise detection of PEDV, this study initially focused on preparing monoclonal antibodies (McAbs) targeted against the recombinant PEDV S1 protein. Next, we developed a colloidal gold immunochromatographic assay (GICA) utilizing this anti-PEDV S1 protein McAbs. The developed method demonstrated robust specificity and sensitivity in distinguishing PEDV-positive from PEDV-negative samples and exhibited a high degree of concordance with the RT-PCR method when applied to the detection of clinical serum samples.

## MATERIALS AND METHODS

### Cell, virus, and mouse

Vero-81 cells were stored in the laboratory for animal infectious diseases, College of Veterinary Medicine, Hebei Agricultural University. It was cultured in Dulbecco’s modified Eagle medium (DMEM, Thermo Fisher Scientific Inc.) containing 10% fetal bovine serum (FBS, Sigma Aldrich, St. Louis, Missouri, USA) and 1% penicillin-streptomycin solution (Procell Life Science & Technology Co., Ltd, China). The PEDV CV777 (GenBank Accession No. AF353511.1) strain and PEDV QY/2016 (QY) strain (GenBank ID MH244927) proliferate and titrate in Vero-81 cells. Three mice were purchased from Beijing Vital River Laboratory Animal Technology Co., Ltd. All animal experiments have been approved by the Experimental Animal Ethics Committee of Hebei Agricultural University.

### Expression and purification of PEDV S1 protein

The pET28a-PEDV S1 plasmid was transformed into *Escherichia coli* BL21 (DE3) cells, followed by induction with 0.5 mM isopropyl β-D-1-thiogalactoside (IPTG) at 35°C for 5 h in Luria-Bertani (LB) medium. The induced expression products were purified by the Ni agarose His affinity chromatography column, and the expression of recombinant protein was analyzed by SDS-PAGE. The purified protein was dialyzed in urea PBS buffer (pH 9) and analyzed by Western blotting using McAbs targeting His-labeled proteins. The protein concentration was determined using the A280 absorption value of NanoDrop 2000 (Thermo Fisher Scientific Inc.).

### Preparation of monoclonal antibodies against PEDV S1

Three female BALB/c mice aged 6–8 weeks were subcutaneously injected with a 1:1 mixture of 50 μg S1 protein and Freund’s complete/incomplete adjuvant (Sigma Aldrich, USA). Blood samples were collected 14 days after the third immunization. The titer of S1-specific antibodies in the serum was determined by indirect ELISA. The mouse with the highest serum titer was selected for intraperitoneal injection of 150 μg S1 protein for booster immunization and was selected for the preparation of hybridoma cells. When the hybridoma cells covered more than one-third of the well bottom area, the supernatant was collected. The supernatant was then tested by ELISA to identify positive clones. Positive hybridoma cell lines were diluted through limiting dilution to obtain a monoclonal cell line that stably produced antibodies. Immunofluorescence assay (IFA) was used to further verify the reactivity of the McAbs against the PEDV S1 protein. The 10-week-old mice were pre-stimulated with 500 μL liquid paraffin wax and injected intraperitoneally with 0.5 mL positive hybridoma cells 10 days later. Mouse abdominal ascites was collected and purified by the ammonium caprylate sulfate method.

### Identification of monoclonal antibodies

#### Western blotting analysis

The immunogenicity and specificity of McAbs were characterized through Western blot analysis using lysates of Vero cells infected with PEDV CV777. Briefly, proteins were transferred to a 0.45 μm PVDF membrane (Thermo Fisher Scientific, USA), and the membrane was blocked with TBS buffer containing 5% skim milk and 0.1% Tween 20 at room temperature for 2 h. Then, the membrane was incubated with purified ascites fluid (primary antibody) at 4°C overnight. Then, the membrane was washed 3 times, and the goat anti-mouse IgG-HRP antibody (Beijing Kangwei Century Biotechnology Co., Ltd.) was added as the secondary antibody at room temperature for 1.5 h. The membrane was incubated with ECL solution (Shanghai Yeasen Biotechnology Co., Ltd.) for 1 min, and the image was captured using a luminescence image analyzer (Shanghai Bilintian Biotechnology Co., Ltd.).

#### Indirect immunofluorescence assay

To evaluate whether monoclonal antibodies can specifically bind to naturally infected viruses, an IFA was performed. Vero cells were placed on 96-well cell culture plates and infected with PEDV CV777 strain (10^7.29^ TCID_50_/mL, MOI = 0.2) or PEDV QY/2016 (10^6.38^ TCID_50_/mL, MOI = 0.2). After 36 h, each well was added with anhydrous methanol 50 μL, fixed at −20°C for 10 min, and then 2% BSA 50 μL, closed at 37°C for 1 h, and each well was added with ascites 50 μL (1:50) and incubated at 37°C for 1 h. Meanwhile, uninfected Vero cells were used as a negative control. The second antibody was FITC-goat anti-mouse IgG (1:250), incubated at 37°C for 45 min, and washed with PBS 3 times. The cells were simultaneously stained using DAPI, observed under an inverted fluorescence microscope, and photographed.

### Affinity analysis of monoclonal antibodies

The affinity of monoclonal antibodies was analyzed by noncompeting enzyme immunoassay. The microplate was coated with 0.1 µg/mL and 0.2 µg/mL antigens. The antibody concentration was diluted from 20 µg/mL in double series to 0.156 mg/L, and the OD_450_ value was determined by indirect ELISA. The formula for the dissociation constant is *K* = (*n* − 1)/2(*n*(6)*t* − [Ab]*t*), *n* = (21)*t*/[Ag′]*t*, where [Ag]*t* and [Ag′]*t* represent different antigen concentrations and [Ab]*t* and [Ab′]*t* represent corresponding antibody concentrations, respectively.

#### Preliminary analysis of monoclonal antibody recognition antigen epitopes

The antibody superposition test was adopted. By calculating the ratio of the superposition of the two antibodies, it was determined whether the antigenic sites recognized by these monoclonal antibodies were the same. The specific steps are as follows. The saturated dilution antibody was combined in pairs as the primary antibody, and the goat anti-mouse antibody was labeled by HRP as the secondary antibody. The additive index (AI) value of the two monoclonal antibodies was calculated by the standard formula. The formula is as follows: AI = (A1.2 − A1)/A2 × 100% (A1 indicates the OD value of the monoclonal antibody; A2 indicates the OD value of another monoclonal antibody. A1.2 represents the combined OD_450_ value of two monoclonal antibodies). Criteria: When the absolute value of AI is less than 10%, the two monoclonal antibodies identify the same antigen site; when the absolute value of AI is greater than 10%, the two monoclonal antibodies identify different antigenic sites.

### Preparation of gold-labeled McAbs

#### Colloidal gold labeling of McAbs

We followed the methods of Yin et al. (21): 99 mL of deionized water and 1 mL of 1% chloric acid solution were added to a clean triangular bottle, which was then placed on a thermostatic magnetic mixer and stirred to boil. Then, 1.8 mL of 1% trisodium citrate solution was added into the boiling deionized water, and the solution was left for 8 min to turn wine red in color. After the reaction, the solution was cooled to room temperature, filtered through a 0.22 μm membrane, sterilized, and then stored at 4°C for future use. The 3 mL colloidal gold suspension was mixed up and down, 30 μL monoclonal antibody with a concentration of 1 mg/mL was added and mixed in a shaking table for 45 min in the dark and then sealed with 10% BSA solution until its final concentration reached 1%. The mixing was continued for 45 min, and the mixture was allowed to stand at 4°C for 2 h. The mixture was then centrifuged at a speed of 12,000 r/min for 30 min at a temperature of 4°C, followed by careful removal of the supernatant. The sediment is blown and mixed with 300 μL double-steamed water containing 0.05% Tween-20, 4% trehalose, and 0.5% BSA, filtered by using a 0.45 μm filter membrane, and stored at a temperature of 4°C.

#### Screening of the strip-paired McAbs

Two McAbs with the highest binding affinity to the PEDV S1 protein were selected. The following steps were performed. The two monoclonal antibody strains were adjusted to 1 mg/mL with PBS buffer and imprinted on the T-line position. The gold-labeled antibody was added dropwise onto the conjugate pad. Then, the sheep anti-mouse IgG was diluted to the same concentration and imprinted on the C-line position of the quality control line. Subsequently, the PEDV S1 protein was diluted 1:20 times with PBS and dropped into the sample hole, and the results were observed within 10–15 min. The color intensity on the nitrocellulose membrane was used to determine the pairing of two specific antibodies.

#### Assembly and criterion of the test strip

The sample pad, bonding pad, nitrocellulose (NC) membrane, absorption pad, and backplane were assembled in sequence. The NC film was affixed to the center of the PVC bottom plate, the binding pad and the absorbent pad were located at both ends of the bottom plate, and the two were superimposed on the NC film for about 2 mm. The gold label pad is affixed between the NC film and the binding pad. After compaction, we use a strip cutter to cut the test board into a 5-mm-wide test strip. The NC membrane was spotted with mAb-H6 as the detection line and sheep anti-mouse IgG as the control line. The sample to be tested is dripped onto the test strip. If the sample to be tested contains PEDV, PEDV first binds with the colloidal gold-labeled antibody to form a complex, which can be captured by the antibody on the T-line, and gold particles aggregate to form a red band. When the complex reaches the C-line, it can be captured by sheep anti-mouse IgG, and thus, a red band will reappear. If the sample to be tested does not contain PEDV, the antibody does not react on the T-line, and the complex only shows a red band at the C-line.

### Evaluation of PRV rapid test strips

#### Specificity analysis of the test strip

To evaluate the specificity of the test strips, PEDV QY-, TGEV-, PCV2-, CSFV-, PRV-, and PRRSV-positive specimens were detected with the test strips. Negative controls were represented by media such as PBS and DMEM. All samples were diluted 1:5 times.

#### Sensitivity analysis of test strips

To evaluate the sensitivity of the test strip, the PEDV QY virus at 10^6.38^ TCID_50_/mL was diluted in a series of 1:10, 1:100, 1:1,000, and 1:10,000 using the sample diluent. Then, 50 μL of each dilution was added to the sample hole. According to the optimal color development time, the lowest detection limit of the S1 protein was observed. This detection limit was calculated by dividing the titer of the PEDV QY strain by the dilution factor and then multiplying by the volume applied (50 μL).

#### The coincidence rate of the test strip

To evaluate the clinical practicability of the colloidal gold strip, 51 clinical samples were diluted 1:5 times and tested by the strip and reverse transcription PCR. The coincidence rate was compared according to the detection results of the two methods. The polymerase chain reaction (PCR) procedure was as follows: initial denaturation at 94°C for 3 min; 35 cycles of 94°C for 45 s, 54°C for 30 s, and 72°C for 45 s, followed by an extension at 72°C for 10 min. Finally, 1.5% agarose gel electrophoresis was used for identification.

## RESULTS

### Expression and purification of recombinant PEDV S1 protein

**Fig 1:**
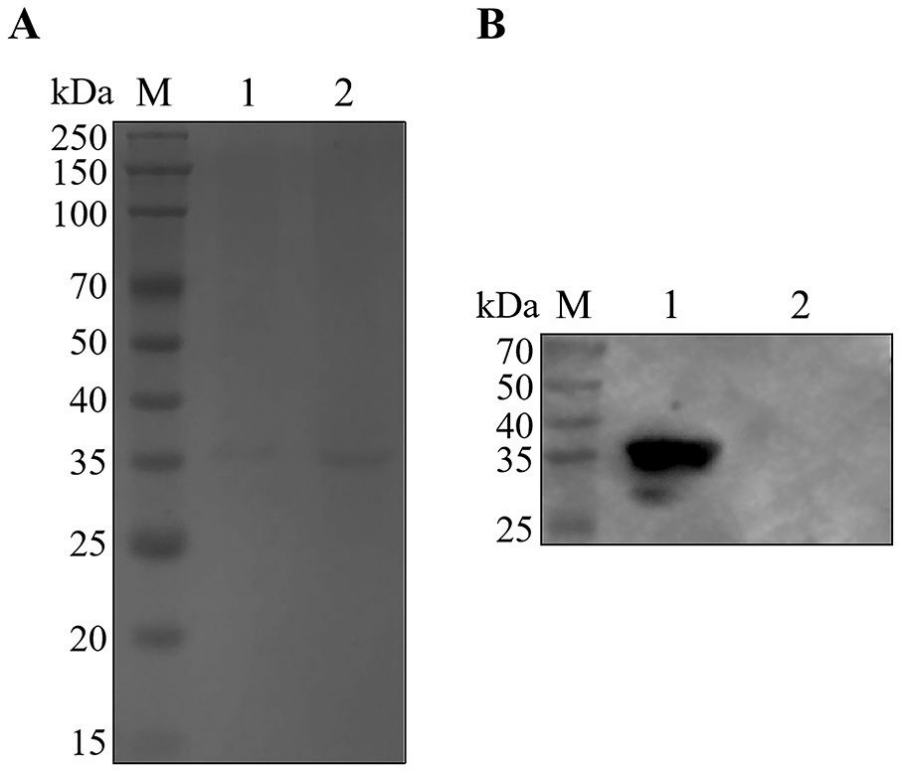
Expression and purification of the recombinant S1 protein of porcine epidemic diarrhea virus (PEDV). (**A**) Expression and purification of PEDV S1 protein. Lane M, protein marker; lanes 1 and 2, purified protein. (**B**) Western blotting identification of the PEDV S1 protein. Lane M, protein marker; lane 1, purified protein; lane 2, empty carrier protein pET28a.

The PEDV S1 recombinant protein was successfully expressed in the bacterial lysate and purified by high-affinity chromatography employing a His tag. As shown in Fig. 1A, the purified PEDV S1 recombinant protein displayed the expected molecular weight. Western blot analysis confirmed that the S1 recombinant protein could react with anti-His tag antibody (HRP) (Fig. 1B).

### Screening and identification of McAbs against PEDV S1 protein

**Fig 2:**
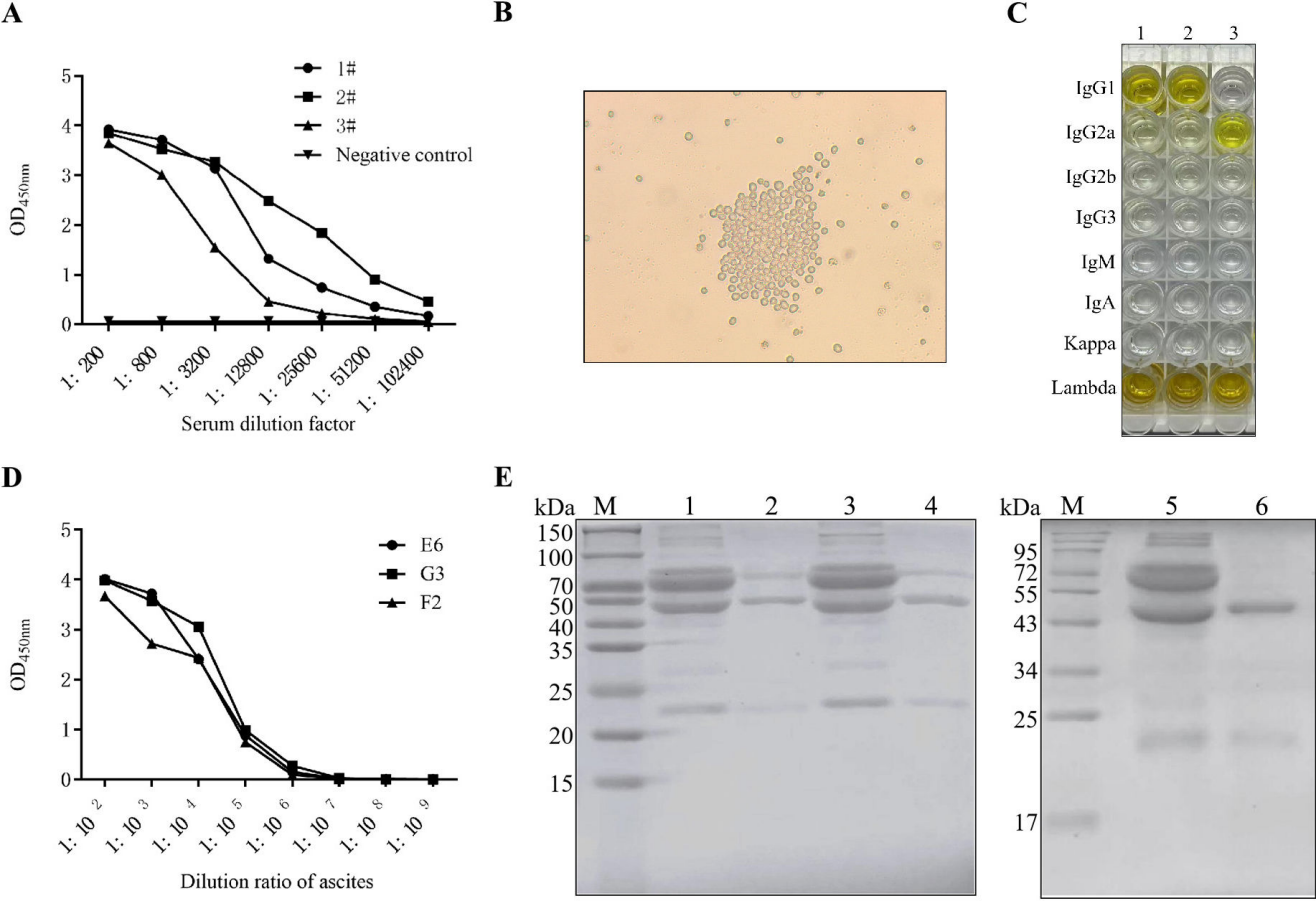
Screening of McAbs. (**A**) Antibody titers in mouse serum. (**B**) Positive hybridoma cell line. Three positive hybridoma cell lines were selected and named as H6, G3, and F2. (**C**) Isotyping of H6, G3, and F2 McAbs. (**D**) Monoclonal antibody ascites titer. The ascites of H6, G3, and F2 McAbs were obtained by the *in vivo* induced ascites method, and their titers were detected by ELISA. (**E**) Purified McAbs H6, G3, and F2 were identified by SDS-PAGE. Lane M, protein marker; lane 1, McAbs H6 before purification; lane 2, purified McAbs H6; lane 3, McAbs G3 before purification; lane 4, purified McAbs G3; lane 5, McAbs F2 before purification; lane 6, purified McAbs F2.

The results are shown in Fig. 2A; the serum antibody titers of mice 1 and 2 both reached 1:102,400, indicating that the effect of immunizing mice with recombinant S1 protein is effective. After cell fusion, a total of 3 positive hybridoma cell lines (H6, G3, and F2) were screened and proliferated *in vitro* (Fig. 2B). To gain a more comprehensive understanding of the characteristics of the McAbs, we used the mouse monoclonal antibody isotyping ELISA kit (Biodragon, Beijing) following the manufacturer’s instructions to determine the subtypes of McAbs. The optical density reading at 450 nm indicated that both McAbs were identified as kappa. The McAbs H6 and G3 belonged to the IgG1 subclass, while mAb F2 belonged to the IgG2a subclass (Fig. 2C). As shown in Fig. 2D, the titers of the three ascites antibodies detected by indirect ELISA all reached 1:10^6^. Purified ascites consisted of heavy and light chains, with the heavy chain exhibiting a molecular weight twice that of the light chain, as shown in Fig. 2E.

### The characteristics of McAbs

**Fig 3:**
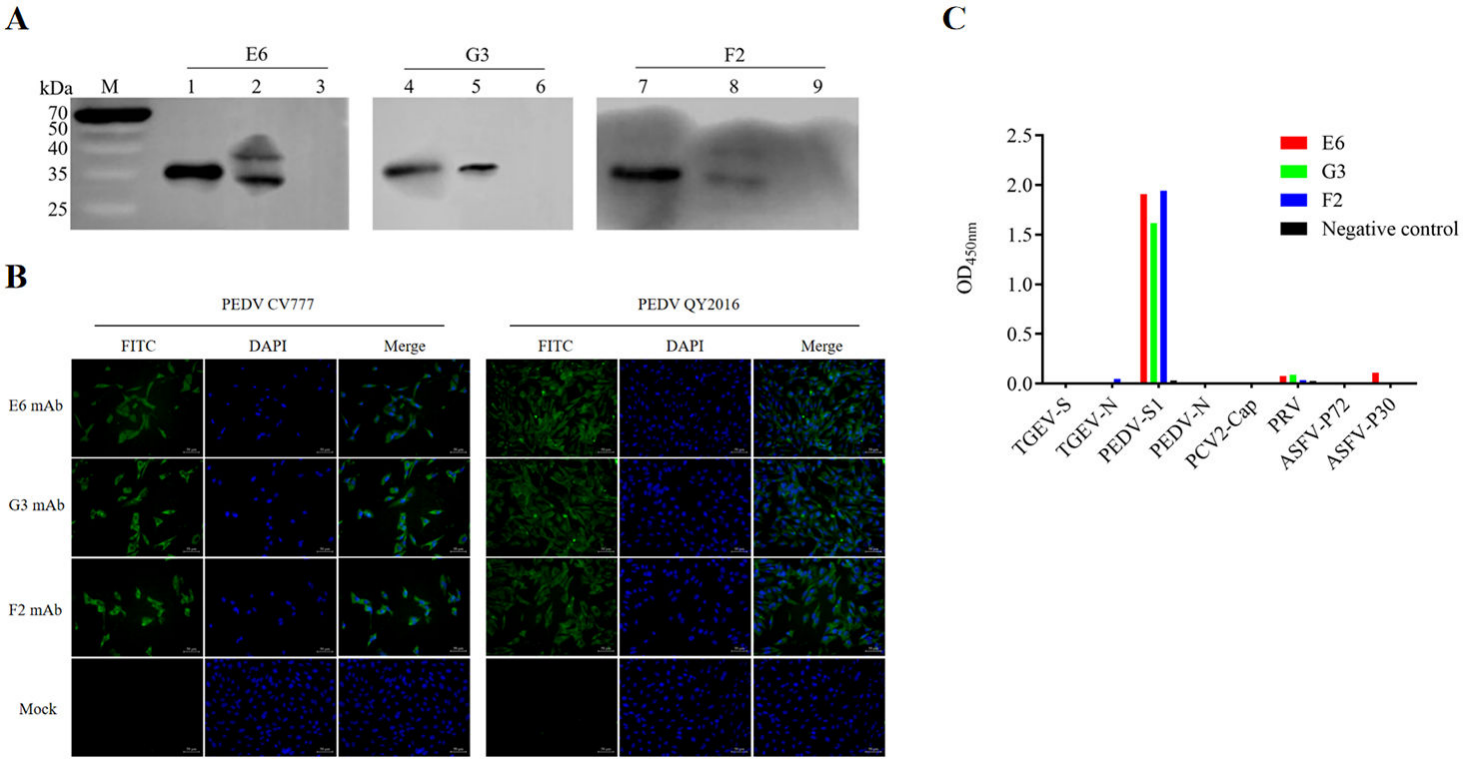
Identification of McAbs. (**A**) McAbs H6, G3, and F2 were specifically bound to the PEDV CV777 strain. Lanes 1, 4, and 7, purified S1 protein; lanes 2, 5, and 8, Vero cells infected with the PEDV CV777 strain; lanes 3, 6, and 9; uninfected Vero cells. (**B**) Vero cells were infected with PEDV strain CV777, and the reactivity of McAbs H6, G3, and F2 against PEDV was confirmed by immunofluorescence assay (IFA). (**C**) The cross-reaction of McAbs H6, G3, and F2 was detected by indirect ELISA.

**Fig 4:**
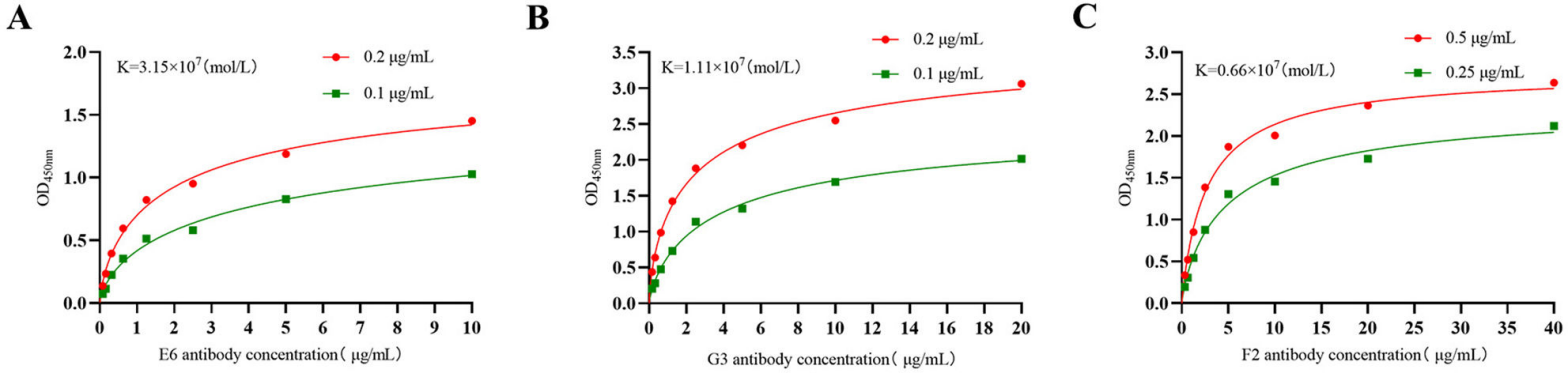
The relative affinity of McAbs. (**A**) The relative affinity of McAbs H6. (**B**) The relative affinity of McAbs G3. (**C**) The relative affinity of McAbs F2.

The reactivity of the monoclonal antibody was assessed by Western blot analysis. As presented in Fig. 3A, bands at the expected 38 kDa size were observed, confirming the interaction between ascites from three monoclonal antibody strains and the recombinant S1 protein. Additionally, these antibodies successfully bind to the PEDV CV777 strain. Conversely, no bands of the expected size were detected when the ascites were tested with the empty carrier protein. To evaluate the binding activity of McAbs H6, G3, and F2 against PEDV, an IFA was performed to analyze their binding capacity to PEDV CV777-infected cells and PEDV QY-infected cells. As shown in Fig. 3B, the IFA demonstrated that all three McAbs generated in this study had strong reactivity. Furthermore, the indirect ELISA results presented in Fig. 3C confirmed that the McAbs specifically reacted with the PEDV S1 protein without exhibiting any cross-reactivity with other protein antigens. The affinities of the three McAbs were 3.15 × 10^7^ mol/L (H6) (Fig. 4A), 1.11 × 10^7^ mol/L (G3) (Fig. 4B), and 0.66 × 10^7^ mol/L (F2) (Fig. 4C), respectively. As presented in Table 1, the antigen index value for F2 was more than 10% higher compared to the other two mAb strains, suggesting that F2 recognizes a distinct antigenic site. Conversely, the antigen index values between H6 and G3 McAbs were less than 10%, indicating that H6 and G3 recognize the same antigen site.

**TABLE 1:** The epitopes identified by mAb H6, G3, and F2 were determined by superimposed ELISA^*a*^

	McAbs	H6	G3	F2
OD_450_ value	H6	1.897	2.129 (6.22%)	2.786 (28.23%)
	G3	1.967 (3.48%)	2.011	2.825 (25.85%)
	F2	3.248 (42.90%)	3.336 (42.08%)	3.149

^*a*^ The cutoff value of the AI value was 10%, AI value >10% was considered to recognize different epitopes, and AI value <10% was considered to recognize the same epitope.

### Establishment and optimization of a rapid detective immunochromatographic strip

**Fig 5:**
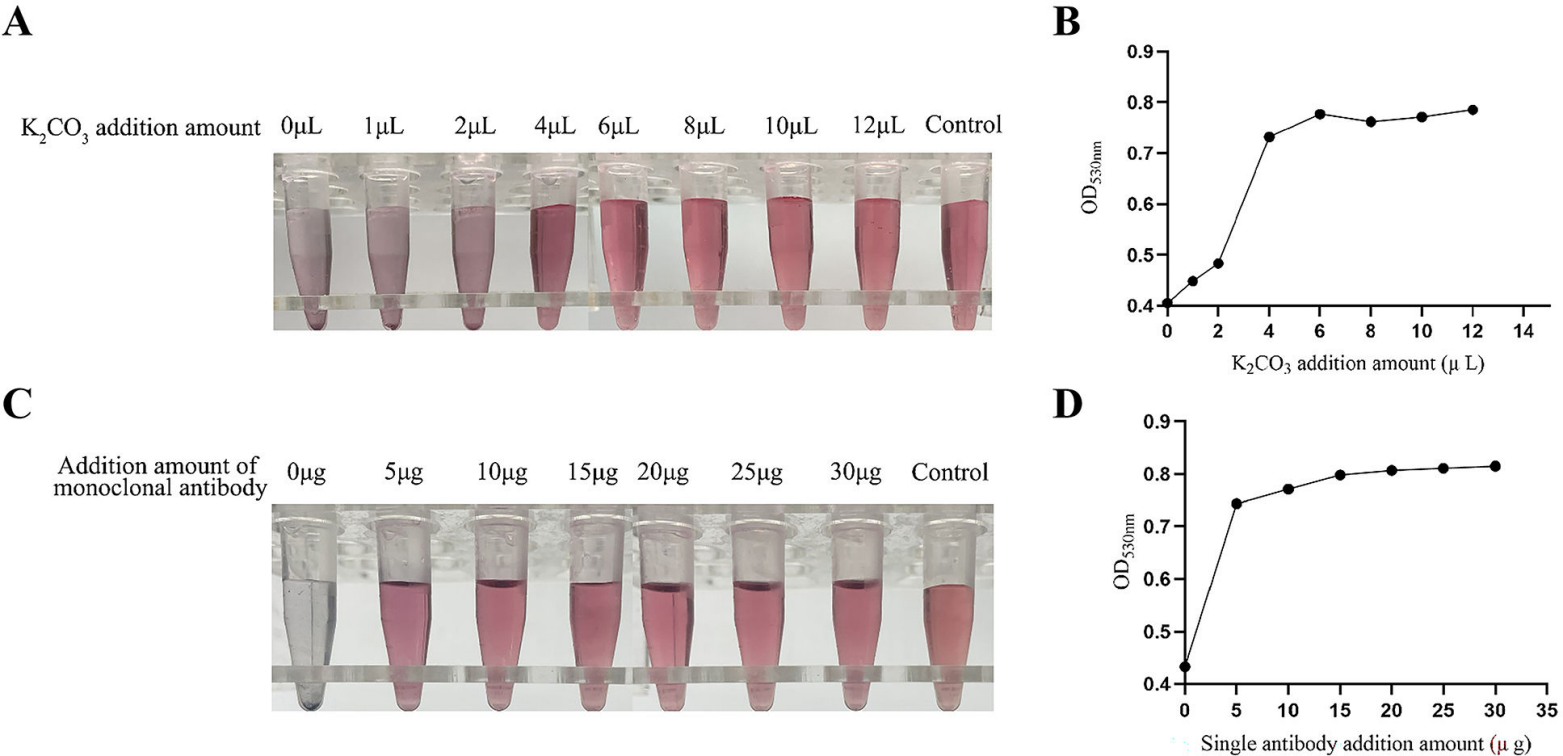
Optimization of the optimal pH value and the optimal addition amount of colloidal gold-labeled antibodies. (**A and B**) The optimal pH value of the colloidal gold-labeled antibody was optimized. (**C and D**) The optimal addition amount of the colloidal gold-labeled antibody was optimized.

**Fig 6:**
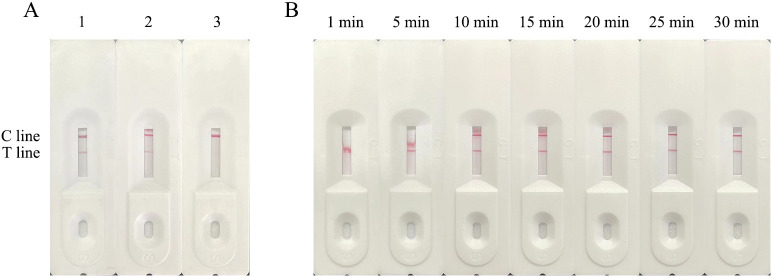
Pairing of monoclonal antibodies and optimization of the optimum color development time of test strips. (**A**) Lane 1, H6 paired with F2; lane 2, G3 paired with F2; lane 3, blank control. (**B**) The optimal color development time of the test strip.

**Fig 7:**
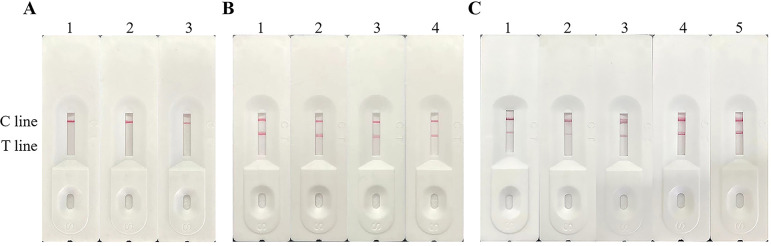
Optimal antibody concentration and coating solution optimization of the quality control line and detection line. (**A**) The concentrations of sheep anti-mouse IgG antibody were 1 mg/mL (lane 1), 0.5 mg/mL (lane 2), and 0.25 mg/mL (lane 3). (**B**) The antibody concentrations were 2.5 mg/mL (lane 1), 2 mg/mL (lane 2), 1.5 mg/mL (lane 3), and 1 mg/mL (lane 4). (**C**) Lane 1, PBS buffer solution; lane 2, PBS buffer containing 3% methanol; lane 3, Tris-HCl buffer containing 4% sucrose, 0.85% NaCl, and 0.1% Tween-20; lane 4, Tris-HCl buffer containing 2% sucrose, 0.85% NaCl, and 0.05% Tween-20; lane 5, Tris-HCl buffer containing 2% sucrose, 0.85% NaCl, and 0.025% Tween-20.

By observing the color of the liquid in the tube and the OD530 nm value, when the amount of monoclonal antibody added was 15 ug, the point on the curve corresponding to the OD530 nm was closest to the *X*-axis (Fig. 5A and B). As demonstrated in Fig. 6A, antibody pairing experiments indicated that the mAb-F2 as the capture antibody and mAb-H6 as the detection antibody showed optimal performance according to the color development in this assay. Furthermore, both the T line and the C line had clearly shown bands after 10 min, confirming that the optimal color development time for the test strip was 10 min (Fig. 6B). When the concentration of sheep anti-mouse IgG was 1 mg/mL and the concentration of the H6 antibody was 2.5 mg/mL, the control line and the detection line of the test strip were the clearest (Fig. 7A and B). Furthermore, as illustrated in Fig. 7C, the antibody test strip was diluted with Tris-HCl buffer containing 2% sucrose, 0.85% NaCl, and 0.05% Tween-20. This dilution produced the most distinct bands on both the detection and quality control lines.

### Specificity analysis of test strips

**Fig 8:**
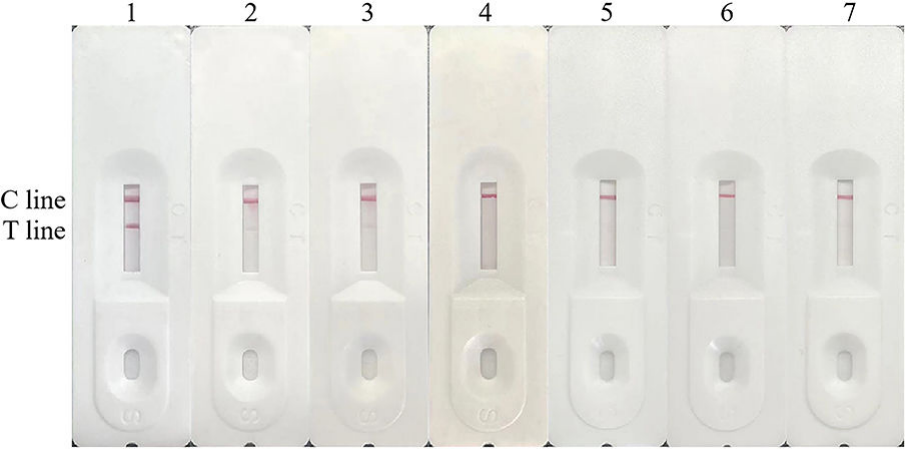
Specificity evaluation of the strip. Lane 1, PEDV CV777; lane 2, TGEV; lane 3, PCV2; lane 4, PRV; lane 5, PRRSV; lane 6, CSFV; lane 7, negative control.

To assess the specificity of the test strips, sera from PEDV-, TGEV-, PCV2-, PRV-, PRRSV-, CSFV-, and PEDV-negative samples were utilized. As shown in Fig. 8, only when detecting PEDV-positive sera, the T line and C line of the PEDV rapid test strip were colored, and TGEV-, PCV2-, PRV-, PRRSV-, CSFV-, and PEDV-negative sera showed T line no color and C line color development results, indicating that the PEDV rapid test strip had good specificity.

### Sensitivity analysis of test strips

**Fig 9:**
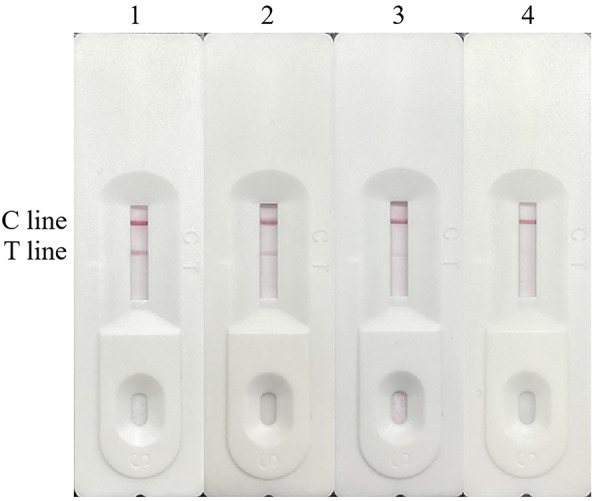
Sensitivity evaluation of the strip. Lanes 1 to 3, detection of PEDV CV777 in different dilutions; lane 4, negative control.

Serial dilutions of the PEDV QY strains ranging from 10^6^ to 10 TCID_50_/mL were used to determine the sensitivity of the strip. As shown in Fig. 9, the results indicate that when the virus was diluted to 1:100, red bands remained visible at the detection line. However, after further dilution to 1:1,000, the red bands disappeared. This suggests that the minimum detection limit for PEDV on the test strip is 1.2 TCID_50_/μL.

### The coincidence rate of the PEDV rapid test strips

A total of 51 sera samples from pigs were detected using both the PEDV rapid test strip and RT-PCR. As presented in Table 2, 30 samples were found positive for PEDV by the RT-PCR, and 28 samples were found positive for PEDV rapid test strips. Based on the detection results of the RT-PCR detection method for PEDV, the coincidence rate of PEDV rapid test strips was 96.1%.

**TABLE 2:** Comparison of the strip with reverse transcription PCR for detection of clinical piglet serum samples

Detection method	Positive (copy)	Negative (copy)	Positive rate（%）	Coincidence rate（%）
Colloidal gold immunoassay test strip	28	23	54.9	96.1
Reverse transcription PCR	30	21	58.8	

## DISCUSSION

The high morbidity and mortality associated with PEDV in piglets facilitate PEDV’s rapid dissemination within swine populations, resulting in substantial economic losses to the global swine industry (22, 23). Similar to other coronaviruses, the S glycoprotein of PEDV serves as the primary target of the immune response, facilitating viral attachment, receptor binding, membrane fusion, and subsequent viral entry into the host cell (24). Notably, the S1 subunit of the S protein plays a pivotal role in recognizing and binding to cellular receptors, and it is also a crucial determinant of PEDV virulence (25). Given the significant impact of PEDV, prophylactic immunization strategies primarily focus on vaccinating pregnant sows to confer passive immunity to piglets. However, the considerable genetic variability of PEDV presents substantial challenges for vaccine development, and currently, no universally efficacious and safe vaccine is available to counteract the threats posed by this virus (26). Consequently, there is an urgent need to develop effective vaccines and immunodiagnostic techniques to formulate novel prevention and control strategies.

Timely monitoring and rapid diagnosis of PEDV infections constitute a crucial approach to the prevention and control of PED. PCR is typically regarded as the gold-standard method for laboratory detection, with RT-PCR and quantitative RT-PCR (qRT-PCR) offering higher sensitivity (27). Currently, ELISA is the most prevalent technique for monitoring PEDV antibody titers, including indirect ELISA protocols established for the detection of IgG, IgA, and sIgA antibodies against PEDV (28, 29). However, these diagnostic methods are time-consuming, necessitate skilled personnel, and are predominantly confined to laboratory settings. Moreover, in resource-poor countries, the pig farming industry faces issues such as a weak epidemic prevention system, a shortage of professionals, and a lack of detection resources. And due to “delayed testing and slow response,” PEDV often leads to large-scale spread, resulting in huge economic losses. To facilitate rapid, on-site detection and the field application of antigen detection, this study introduces an easy-to-operate and sensitive immunochromatographic test strip for utilizing anti-PEDV S1 monoclonal antibodies. Colloidal gold test strips, as an immediate detection technology, have core advantages such as extremely simple operation, no need for complex equipment, rapid result output, controllable cost, and intuitive reading. It perfectly addresses the pain points of resource-poor countries in epidemic prevention, namely, lack of personnel, equipment, and funds, providing a low-cost solution for grassroots prevention of PEDV.

The expression of viral proteins in eukaryotic cells exhibits biological activity but is often costly (30). In contrast, prokaryotic expression systems enable rapid production of large quantities of recombinant proteins using simple and inexpensive bacterial cell cultures (31). However, due to the large molecular weight of the full-length S protein, direct expression in a prokaryotic system is challenging. Consequently, in this study, we elected to express the S1 subunit of the S protein in *E. coli* BL21 (DE3). After purification and concentration, the recombinant S1 protein with high purity and good reactivity was obtained by SDS-PAGE and Western blotting analysis.

Many molecular diagnostic methods have been reported and are used in laboratories for highly sensitive and accurate virus detection. These methods play a significant role in pathogen molecular epidemiological studies (32–36). To develop a highly specific detection method for PEDV, we generated three McAbs targeting the purified S1 protein, H6, G3, and F2. Functional analysis showed that the three monoclonal antibodies were suitable for ELISA, Western blotting, and IFA detection. The remaining three McAbs can be used for fundamental research in the field of pathogen detection, antigen purification, disease diagnosis, and prevention for PEDV. In addition, the antigen epitopes recognized by F2 were different from those recognized by H6 and G3; although we did not further identify their fine epitopes, we established a colloidal gold immunochromatographic test strip method for PEDV detection. The McAbs H6 and F2 were selected as detection and capture antibodies, respectively, in the sandwich detection mode for the immunochromatographic test strip. This test strip can only detect PEDV without cross-reactivity with other porcine viruses (TGEV, PCV2, CSFV, PRV, and PRRSV). In addition, the test strip achieves high sensitivity with a detection limit of 1.2 TCID_50_/μL. This sensitivity is significantly higher than that reported previously (32). Subsequently, a comparative analysis was conducted on 51 clinical serum samples using both the test strip and RT-PCR. The findings demonstrated a positive coincidence rate of 54.9%, a negative coincidence rate of 58.8%, and an overall agreement rate of 96.1% between the two methods.

In this study, a high-purity S1 recombinant protein was obtained and used to immunize mice, resulting in the production of highly sensitive and specific McAbs F2 and H6. These McAbs were then used in the development of an immunochromatographic test strip for rapid detection of PEDV. This diagnostic method, leveraging paired labeled McAbs for antigen detection, demonstrates high sensitivity and specificity, making it a valuable substitute for RT-PCR in the clinical diagnosis of PEDV.

## Data Availability

The data sets used and analyzed during the current study are available from the corresponding author on reasonable request.
